# CARING FOR ONE, TWO, OR THREE GENERATIONS? PREVALENCE OF VARIOUS CAREGIVING ROLES AND CAREGIVER CHARACTERISTICS

**DOI:** 10.1093/geroni/igae098.4138

**Published:** 2024-12-31

**Authors:** Hua Zan, Loriena Yancura, Melissa Barnett

**Affiliations:** University of Hawaii at Manoa, Honolulu, Hawaii, United States; University of Hawaii at Manoa, Honolulu, Hawaii, United States; University of Arizona, Tucson, Arizona, United States

## Abstract

With increasing diversification in household composition and living arrangements over recent decades, the roles of family caregivers have become more complex, sometimes requiring individuals to assume multiple caregiving responsibilities such as caring for both grandchildren and older parents. While caregiving can be a rewarding experience, it can negatively affect caregiver well-being, especially for those providing high-intensity care. Given that family caregivers are the backbone of our care system, it is crucial to understand who these caregivers are and their health and wellbeing. This study provides a profile of U.S. intergenerational caregivers using data from 2018 RAND Health and Retirement Study (HRS) family files and HRS core public files. We estimate the prevalence of four different caregiver types (i.e., caring for older parents only, spouses only, grandchildren only, and those filling more than one caregiving role) overall and by socio-demographic characteristics. We also compare the socio-demographic characteristics and health outcomes (e.g., self-reported health and depression) of different caregiver types to non-caregivers. Our results showed that one in three adults age 50 or older were caregivers, the majority of which only cared for one generation: grandchildren, spouses, or older adults. Approximately 14% of caregivers cared for multiple types of family members. Caregiver profiles and health outcomes vary by caregiver type. For example, spousal caregivers had the highest depression score compared to individuals only caring for grandchildren or older parents and those filling more than one caregiving role. This knowledge is essential for establishing a foundation for targeted interventions to support caregivers.

